# Continuity of Prescription Medication Use Among Adults Leaving State Prison

**DOI:** 10.1001/jamanetworkopen.2024.61982

**Published:** 2025-02-27

**Authors:** Laura Dague, Cici McNamara, Ryan Westergaard, Kevin A. Look, Marguerite Burns

**Affiliations:** 1 Bush School of Government and Public Service, Texas A&M University, College Station; 2 School of Economics, Georgia Institute of Technology, Atlanta; 3 School of Medicine and Public Health, University of Wisconsin–Madison, Madison; 4 School of Pharmacy, University of Wisconsin–Madison, Madison

## Abstract

**Importance:**

Identifying prescription needs of adults returning from prison can inform intervention design to mitigate high morbidity and mortality during reentry to the community.

**Objective:**

To characterize the prevalence and factors associated with continuity of medication use for treatment of chronic illness among adults returning to the community from prison.

**Design, Setting, and Participants:**

This retrospective cohort study linked administrative records and prescription claims from the Wisconsin Department of Corrections and the Medicaid program. The study included all adults (aged 18-64 years) released from a Wisconsin state correctional facility between April 1, 2015, and June 30, 2017, after incarceration of at least 31 days who enrolled in Medicaid within the month of release. Analyses were conducted between May 2022 and May 2024.

**Exposure:**

Receipt of a prescription medication commonly used for treatment of certain chronic conditions during the last 3 months of incarceration.

**Main Outcomes and Measures:**

The primary outcome was medication continuity, which was defined as having a prescription medication claim in the first 6 months after release that was within the same chronic condition therapeutic class as the medication received during the last 3 months of incarceration.

**Results:**

This cohort study included 13 792 individual releases representing 12 960 individuals from the Wisconsin state prison system who immediately enrolled in Medicaid upon release. These individuals had a mean (SD) age of 35.5 (10.5) years; 89.5% were male. In terms of race, 38.5% of individuals were Black and 57.2% were White. For one-third of individual Medicaid-enrolled releases (4302 [31.2%]), the individual received a prescription medication for a chronic condition. Prescription continuity was observed for 51.7%, with variability across drug class ranging from less than 20.0% to 71.6%. Classes with the highest continuity (eg, thyroid agents) were also those that treated serious chronic conditions, although gaps between realized and clinically recommended care remained. Continuity varied by race, sex, and age. Individuals with continuity were more likely to have had an outpatient visit in the 6 months following release than those without continuity (93.9% vs 55.6%; *P* < .001) despite similar Medicaid enrollment.

**Conclusions and Relevance:**

In this study, prescription continuity between prison release and the community was limited, even for drugs with high clinical need and relevance for population health and especially among individuals who did not receive outpatient care in the 6 months post incarceration. These findings suggest that effective transitional care interventions may need to include increased emphasis on connecting with clinicians.

## Key Points

**Question:**

How often do adults returning to the community from prison continue to access prescription medications, and what factors are associated with medication continuity?

**Findings:**

In this cohort study of 13 792 releases representing 12 960 individuals, 52% of adults released from Wisconsin state correctional facilities who immediately enrolled in Medicaid maintained continuity of medications commonly used for treatment of chronic illnesses, with substantial variability across drug classes. Those with continuity were significantly more likely to have had an outpatient visit within 6 months post release.

**Meaning:**

These findings highlight the need for improved transitional care interventions to enhance medication continuity among adults reentering the community from prison.

## Teaser

This cohort study examines the prevalence and factors associated with prescription medication continuity among Medicaid-insured adults returning to the community after release from Wisconsin state correctional facilities.

## Introduction

High morbidity and mortality characterize the period of transition following release from incarceration, making access to and continuation of health care during reentry an essential part of supporting a healthy return to the community.^1,2^ Rates of chronic illness among justice-involved adults are substantially higher than those observed in comparable populations without justice involvement, and the likelihood of receiving treatment for many chronic conditions in the community declines relative to during incarceration.^3,4,5,6^ Medication adherence can decrease morbidity, mortality, and health care costs, particularly among patients with chronic diseases.^7,8^ However, both financial and practical barriers to accessing prescription medications upon return to the community have been noted as disruptions to continuity of care by physicians who work with incarcerated and reentering populations.^9^

Increased eligibility for Medicaid, which typically allows access to prescriptions with little or no cost-sharing, may improve access to care among individuals recently released from prison, but access alone may not improve health if other critical barriers remain.^10,11,12^ Federal policy prohibits Medicaid coverage during incarceration, creating practical challenges in care access and coordination at release. The 2023 SUPPORT Act (Substance Use-Disorder Prevention That Promotes Opioid Recovery and Treatment Act) requires the Centers for Medicare & Medicaid Services (CMS) to provide guidance on how states could improve care transitions through Medicaid 1115 reentry waivers.^13^ Eleven states have approved waivers, with 13 pending, and all include minimum requirements for case management, a 30-day supply of prescriptions at release, and postrelease appointment setup assistance.^14^

Limited access to medical records from correctional settings has stymied efforts to identify health care needs before individuals leave prison, and prior efforts to understand medication continuity post incarceration are qualitative, focus on a single disease, or both.^15,16,17,18^ We connected data on medication prescriptions from the carceral period to postrelease claims and identified individuals with likely ongoing medication treatment needs for chronic conditions. We aimed to determine the likelihood of continued prescription fills after release and identify demographic, carceral, and health care utilization factors associated with prescription medication continuity for certain medications commonly used to treat chronic conditions among adults returning to the community from prison.

## Methods

The University of Wisconsin Institutional Review Board approved this cohort study. Informed consent was waived because the research involved no more than minimal risk and could not be carried out without the waiver. This study followed the Strengthening the Reporting of Observational Studies in Epidemiology (STROBE) reporting guideline.

### Data Source

We connected data on medication prescriptions from the carceral period to postrelease claims and identified individuals with likely ongoing medication treatment needs for chronic conditions. We linked person-level administrative data on timing and characteristics of carceral sentences and newly available data on carceral prescriptions from the Wisconsin Department of Corrections to Medicaid enrollment and claims from the Division of Medicaid Services in the Wisconsin Administrative Data Core.^19^

### Study Cohort

The study cohort included adults (aged 18-64 years) who were released to the community from a Wisconsin state correctional facility between April 1, 2015, and June 30, 2017, after incarceration of at least 31 days and who enrolled in Medicaid within 1 month of release and for at least 1 additional month (13 792 releases for 12 960 individuals). All released individuals had access to prison-based Medicaid enrollment assistance and were given a 2-week supply of any needed medications plus a prescription for an additional 30-day supply.^20^ Wisconsin allows adults younger than 65 years with incomes up to the poverty line to enroll in Medicaid.^21^ Additional study sample information is presented in eMethods 1, eFigure 1, and eTable 1 in Supplement 1.

### Medication Continuity

We defined prescription continuity as having at least 1 prescription for certain medications commonly used to treat chronic illness during the last 3 months of incarceration and at least 1 claim within the first 6 months after release in the same medication class. Measure construction details are presented in eMethods 2 and eFigure 2 in Supplement 1. We identified all prescriptions during the last 3 months of incarceration, including drugs that are dispensed over the counter (OTC) in the community, because all medications administered within the correctional setting are prescribed. To identify chronic illness medications, we excluded OTC drugs in the community, those for acute conditions, and those indicated for multiple conditions. Each medication was mapped to its appropriate American Hospital Formulary Service (AHFS) chronic illness therapeutic class (eTable 2 in Supplement 1). The top 25 prescribed medications and those identified as commonly used for treatment of chronic illness are included in eTable 3 in Supplement 1.

We created binary measures indicating a Medicaid prescription claim in the same class within 6 months of release. Aggregation by class, rather than by specific medication, accounts for differing formularies across prison and Medicaid. Additional details are provided in eMethods 2 in Supplement 1. We defined an alternative continuity measure across AHFS classes indicated for hypertension, hypercholesterolemia, diabetes, anxiety or depression, severe mental illness (SMI) (ie, bipolar disorder and schizophrenia), and HIV, due to their specific prevalence or potential for multiple appropriate AHFS classes.

### Other Measures

We measured individual age, race, marital status, education, county of conviction, duration of incarceration, security level of release facility, duration of Medicaid enrollment post release, and indicators for health care use during the 6 months after release (ie, outpatient, emergency department [ED], and inpatient visits). Sex, marital status, race, and education are all self-reported at the time of admission to the correctional facility, and education may have changed by release. Race was included in this analysis because it is associated with the outcome (data on ethnicity were not available). Race is reported as Black, White, or other race (the latter of which includes American Indian or Alaska Native, Asian or Pacific Islander, and unknown race).

### Statistical Analysis

We detailed prescription continuity overall and by class, including exact binomial 95% Clopper-Pearson CIs. We used the Pearson χ^2^ test to compare characteristics between those with and without continuity and estimated the association between these characteristics and continuity using logistic regression, reporting average marginal effects^22^ alongside exact *P* values and *q* values corrected for a false discovery rate using the Benjamini-Hochberg procedure. A threshold of *P* < .05 (2-tailed) was used to assess statistical significance. We examined timing of health care use relative to prescription fill and time to fill for those with and without an outpatient visit in the first 6 months. The unit of analysis was prison release, with certain individuals possibly contributing multiple releases; SEs in the regression analyses are clustered by individual to account for within-person associations. Results controlling for the number of releases and limiting to the first release were very similar (eTable 4 in Supplement 1).

Data analysis was performed with Stata, version 18 (StataCorp LLC). Analyses were conducted between May 2022 and May 2024.

## Results

This study included 13 792 individual releases representing 12 960 individuals. Summary demographic information is provided in eTable 1 in Supplement 1. Among the 13 792 individual Medicaid-enrolled releases, the enrollees had a mean (SD) age of 35.5 (10.5) years; 10.5% were female, 89.5% were male, and 27.0% had less than a high school education. In terms of race, 38.5% were Black, 57.2% were White, and 4.2% were of other race. Most (80.7%) were convicted in a nonrural county, with a mean (SD) duration of 24.5 (33.0) months of incarceration; 52.7% had been incarcerated in medium-security facilities.

Table 1 summarizes continuity of prescription access by AHFS therapeutic class and our aggregate disease categories. Among the 13 792 releases for individuals who enrolled in Medicaid within the month of release, there were 4302 releases (31.2%) in which the individual received a prescription medication during the last 3 months before release for at least 1 chronic condition defined by an AHFS class of interest. Table 1 presents the ranked list aggregated by AHFS class of prescription medications received during the last 3 months of incarceration. At the top of the list, there were 1170 releases (8.5%) in which the individual had a prescription for an atypical antipsychotic during the last 3 months of incarceration, followed by a prescription for angiotensin-converting enzyme inhibitors (1060 [7.7%]) or 3-hydroxy-3-methylglutaryl coenzyme A (HMG-CoA) reductase inhibitors (ie, statins) (1035 [7.5%]). By broad disease category (Table 1), there were 4522 individual releases (32.8%) in which the individual received medication for anxiety or depression, 2592 (18.8%) for hypertension, 1545 (11.2%) for SMI, 1056 (7.7%) for hypercholesterolemia, 572 (4.2%) for diabetes, and 47 (0.3%) for HIV.

**Table 1.** Prescription Continuity Post Release for AHFS Drug Classes and Diseases of Interest^a^

Rank	AHFS drug class or disease class	Individual releases with a fill during the 3 mo before release (N = 13 792)^b^	Individual releases with a fill during the 6 mo post release (n = 4302)^c^
AHFS drug class of interest			
1	Atypical antipsychotics	1170 (8.5) [8.0-9.0]	502 (42.9) [40.0-45.8]
2	Angiotensin-converting enzyme inhibitors	1060 (7.7) [7.2-8.1]	516 (48.7) [45.6-51.7]
3	HMG-CoA reductase inhibitors	1035 (7.5) [7.1-8.0]	552 (53.3) [50.2-56.4]
4	Thiazide diuretics	968 (7.0) [6.6-7.5]	350 (36.2) [33.1-39.3]
5	β-Adrenergic blocking agents	877 (6.4) [6.0-6.8]	408 (46.5) [43.2-49.9]
6	Corticosteroids (EENT)	602 (4.4) [4.0-4.7]	254 (42.2) [38.2-46.2]
7	Central α-agonists	540 (3.9) [3.6-4.2]	180 (33.3) [29.4-37.5]
8	Biguanides	427 (3.1) [2.8-3.4]	258 (60.4) [55.6-65.1]
9	Dihydropyridines	424 (3.1) [2.8-3.4]	194 (45.8) [40.9-50.6]
10	Thyroid agents	342 (2.5) [2.2-2.8]	245 (71.6) [66.5-76.4]
11	Antimania agents	354 (2.6) [2.3-2.8]	129 (36.4) [31.4-41.7]
12	Rapid-acting insulins	299 (2.2) [1.9-2.4]	134 (44.8) [39.1-50.6]
13	Butyrophenones	200 (1.4) [1.3-1.7]	44 (22.0) [16.5-28.4]
14	Anticholinergic agents (CNS)	204 (1.5) [1.3-1.7]	55 (27.0) [21.0-33.6]
15	Sulfonylureas	176 (1.3) [1.1-1.5]	70 (39.8) [32.5-47.4]
16	Angiotensin II receptor antagonists	185 (1.3) [1.2-1.6]	102 (55.1) [47.7-62.4]
17	Leukotriene modifiers	142 (1.0) [0.9-1.2]	48 (33.9) [26.1-42.2]
18	Antimuscarinics	135 (1.0) [0.8-1.2]	39 (28.9) [21.4-37.3]
19	Thiazide-like diuretics	128 (0.9) [0.8-1.1]	32 (25.0) [17.8-33.4]
20	Calcium channel blocking agents, miscellaneous	120 (0.9) [0.7-1.0]	45 (37.5) [28.8-46.8]
21	Antimuscarinics or antispasmodics	83 (0.6) [0.5-0.8]	40 (48.2) [37.1-59.4]
22	Fibric acid derivatives	71 (0.5) [0.4-0.6]	20 (28.2) [18.1-40.1]
23	Antigout agents	65 (0.5) [0.4-0.6]	38 (58.5) [45.6-70.6]
24	α-Adrenergic blocking agents	53 (0.4) [0.3-0.5]	24 (45.3) [31.6-59.6]
25	EENT drugs, miscellaneous	49 (0.4) [0.3-0.5]	NA^d^
Any	Any prescription in AHFS drug class of interest	4302 (31.2) [30.4-32.0]	2223 (51.7) [50.2-53.2]
Disease class			
1	Anxiety or depression	4522 (32.8) [32.0-33.6]	2073 (45.8) [44.4-47.3]
2	Hypertension	2592 (18.8) [18.1-19.5]	1245 (48.0) [46.1-50.0]
3	Severe mental illness	1545 (11.2) [10.7-11.7]	639 (41.4) [38.9-43.9]
4	Hypercholesterolemia	1056 (7.7) [7.2-8.1]	564 (53.4) [50.4-56.4]
5	Diabetes	572 (4.2) [3.8-4.5]	371 (64.9) [60.8-68.8]
6	HIV	47 (0.3) [0.2-0.4]	37 (78.7) [64.3-89.3]
Any	Any prescription in disease class of interest	5923 (43.0) [42.1-43.8]	3019 (51.0) [49.7-52.2]

Abbreviations: AHFS, American Hospital Formulary Service; CNS, central nervous system; EENT, ear, eye, nose, and throat; HMG-CoA, 3-hydroxy-3-methylglutaryl coenzyme A; NA, not available.

^a^ Data are presented as No. (%) of individual releases [95% CI].

^b^ Reflects the number of individual releases from a Wisconsin state correctional facility between April 1, 2015, and June 30, 2017, who received a prescription for a drug in the corresponding class in the 3 months preceding release.

^c^ Reflects the number of individual releases among enrollees who had a Medicaid fill for a drug in the 6 months following release from among those who had received a prescription for a drug in the corresponding class in the 3 months preceding release.

^d^ Group under the sample size disclosure threshold.

The percentage of the study cohort with continuity in any class during the first 6 months after release was 51.7% (Table 1). There was wide variance in continuity by AHFS class, from a low of less than 20.0% (exact percentage suppressed due to sample size disclosure requirements) among individuals receiving miscellaneous ear, eye, nose, and throat medications before release to a high of 71.6% for thyroid agents. Of the top 5 prescribed disease classes before release, just 1 had continuity higher than 50% (HMG-CoA reductase inhibitors, 53.3%). When categorized by disease (Table 1), there was relatively high although incomplete continuity for HIV medications (78.7%), followed by medications for diabetes (64.9%) and hypercholesterolemia (53.4%); fewer than half had continuity for medications for hypertension (48.0%), anxiety or depression (45.8%), or SMI (41.4%).

Table 2 presents means (SDs) by characteristic among those with and without AHFS class continuity, alongside *P* values for the tests of the difference in means. Compared with their counterparts without prescription continuity, individuals with continuity were more likely to be female (19.8% vs 10.9%; *P* < .001) and older (mean [SD], 42.9 [10.5] vs 37.5 [10.6] years; *P* < .001). They were less likely to be single (85.6% vs 89.1%) and more likely to be married or partnered (13.1% vs 8.9%) (*P* < .001). In addition, they were less likely to be Black (34.6% vs 43.0%), more likely to be White (62.0% vs 51.8%), and less likely to be of other race (3.4% vs 5.1%) (*P* < .001). They also were slightly less likely to have less than a high school education (24.8% vs 28.3%) or to be missing education (1.9% vs 2.6%) and more likely to have at least a high school education (73.3% vs 69.2%) (*P* = .008). Those with continuity were less likely to be from a nonrural county of conviction (77.9% vs 80.9%; *P* = .01) and had longer incarceration durations (mean [SD], 36.9 [46.0] vs 31.2 [35.8] months; *P* < .001). Finally, those with prescription continuity were more likely to have been incarcerated at minimum security facilities (43.2% vs 35.1%) and less likely to have been incarcerated at maximum security facilities (6.0% vs 9.7%) compared with those without continuity (*P* < .001). There was no significant difference in the share of the 2223 releases with continuity vs the 2079 releases without continuity who were enrolled in Medicaid for at least 6 months post release (71.3% vs 69.6%; *P* = .22).

**Table 2.** Characteristics Associated With Continuity

Characteristic	Individual releases (N = 4302)^a^		*P* value^b^
	No fill within 6 mo (n = 2079)	Fill within 6 mo (n = 2223)	
Enrolled in Medicaid for ≥6 mo post release	1447 (69.6)	1585 (71.3)	.22
Any outpatient, ED, or hospital visit	1265 (60.8)	2122 (95.5)	<.001
Outpatient	1157 (55.6)	2087 (93.9)	<.001
ED	372 (17.9)	540 (24.3)	<.001
Hospital	109 (5.2)	267 (12.0)	<.001
Sex			
Male	1852 (89.1)	1782 (80.2)	<.001
Female	227 (10.9)	441 (19.8)	
Age at release, mean (SD), y	37.5 (10.6)	42.9 (10.5)	<.001
Marital status			
Single	1852 (89.1)	1904 (85.6)	<.001
Married or partnered	185 (8.9)	292 (13.1)	
Other	42 (2.0)	27 (1.2)	
Race			
Black	895 (43.0)	769 (34.6)	<.001
White	1078 (51.8)	1379 (62.0)	
Other^c^	106 (5.1)	75 (3.4)	
Education			
<High school	588 (28.3)	551 (24.8)	.008
≥High school	1438 (69.2)	1630 (73.3)	
Missing	53 (2.6)	42 (1.9)	
Rural status of county of conviction			
Nonrural	1682 (80.9)	1732 (77.9)	.05
Rural	370 (17.8)	453 (20.4)	
Missing	27 (1.3)	38 (1.71)	
Duration of incarceration, mean (SD), mo	31.2 (35.8)	36.9 (46.0)	<.001
Security status of release facility			
Minimum	730 (35.1)	961 (43.2)	<.001
Medium	1038 (49.9)	947 (42.6)	
Medium/maximum	107 (5.2)	179 (8.0)	
Maximum	201 (9.7)	134 (6.0)	
Jail	NA^d^	NA^d^	

Abbreviations: ED, emergency department; NA, not applicable.

^a^ Data are presented as the mean (SD) for continuous measures and No. (%) of releases for categorical measures. Sample is all individual releases between April 1, 2015, and June 30, 2017 who had at least 1 prescription for a drug in 1 of the 25 drug classes of interest from Table 1 in the 3 months preceding release and who were enrolled in Medicaid in the month of release.

^b^ *P* values for binary and categorical variables were constructed using Pearson χ^2^.

^c^ Includes American Indian or Alaska Native, Asian or Pacific Islander, and unknown race.

^d^ Under the sample size disclosure threshold.

Major differences were present in health care use after release among those with and without prescription continuity. A total of 1265 of 2079 releases (60.8%) with no continuity had an outpatient, emergency, or hospital visit, compared with 2122 of 2223 releases (95.5%) with continuity (*P* < .001). Those without continuity were less likely to have used all types of health care services, with the largest absolute difference in outpatient visits (55.6% vs 93.9%; *P* < .001), followed by hospital visits (5.2% vs 12.0%; *P* < .001) and then ED visits (17.9% vs 24.3%; *P* < .001). eFigure 3 in Supplement 1 includes a summary of estimated differences in continuity by postrelease health care utilization for each disease category and the top 5 AHFS classes along with 95% CIs. With the exception of HMG-CoA reductase inhibitors, all showed higher continuity if they had an outpatient visit within the first 6 months post release, while there were no consistent differences in continuity for emergency and hospital visits.

Table 3 presents the associations between sample characteristics (ie, demographics, carceral, postrelease health care use) and continuity from a logistic regression analysis. Health care use remained associated with continuity conditional on personal and carceral characteristics. Having an outpatient visit in the 6 months following release was associated with a 43.8 (95% CI, 40.8-46.9)–percentage point increase in the likelihood of continuity (*P* < .001). The analogous estimate for having a hospital visit in the 6 months following release was 10.0 (95% CI, 5.0-14.9) percentage points (*P* < .001), but there was no association between ED visits and continuity in the 6 months following release (2.4 [95% CI, −1.0 to 5.8] percentage points; *P* = .16). Sex, age, and race remained associated with continuity.

**Table 3.** Regression-Adjusted Associations Between Prescription Fills Within 6 Months of Release and Individual Characteristics

Characteristic	Percentage point difference relative to baseline (95% CI) (n = 4302 observations)^a^	*P* value	*q* Value^b^
Enrolled in Medicaid for ≥6 mo post release	0.8 (−2.2 to 3.7)	.62	.78
Health care use within 6 mo post release, visit type			
Outpatient	43.8 (40.8-46.9)	<.001	<.001
ED	2.4 (−1.0 to 5.8)	.16	.34
Hospital	10.0 (5.0-14.9)	<.001	<.001
Sex			
Male	1 [Reference]	NA	NA
Female	10.9 (5.7-16.2)	<.001	<.001
Age at release	0.8 (0.7-1.0)	<.001	<.001
Marital status			
Single	1 [Reference]	NA	NA
Married or partnered	4.0 (−0.2 to 8.1)	.06	.20
Other	−13.4 (−28.2 to 1.5)	.08	.21
Race			
Black	−0.4 (−3.4 to 2.6)	.78	.82
White	1 [Reference]	NA	NA
Other^c^	−11.6 (−18.4 to −4.7)	.001	.003
Education			
Less than high school	1 [Reference]	NA	NA
High school or greater	−0.6 (−3.7 to 2.5)	.70	.81
Missing	3.1 (−8.1 to 14.2)	.59	.78
Rural status of county of conviction			
Nonrural	1 [Reference]	NA	NA
Rural	0.2 (−3.2 to 3.6)	.91	.91
Missing	7.0 (−3.8 to 17.9)	.20	.39
Duration of incarceration, mo	0.03 (−0.01 to 0.1)	.10	.23
Security status of release facility			
Minimum	1 [Reference]	NA	NA
Medium	−1.1 (−4.2 to 1.9)	.46	.68
Medium/maximum	−3.7 (−10.6 to 3.3)	.30	.47
Maximum	−2.8 (−7.9 to 2.4)	.30	.47
Jail	−7.2 (−47.3 to 32.9)	.72	.81

Abbreviations: ED, emergency department; NA, not applicable.

^a^ Results of logistic regression of indicator for prescription continuity on individual characteristics. Cells contain marginal effects × 100 with 95% CIs. SEs are clustered at the individual level. Sample is all individual releases between April 1, 2015, and June 30, 2017, who had at least 1 prescription for a drug in one of the drug classes of interest from Table 1 in the 3 months preceding release and who were enrolled in Medicaid in the month of release.

^b^ Computed using the Benjamini-Hochberg procedure.

^c^ Includes American Indian or Alaska Native, Asian or Pacific Islander, and unknown race.

**Figure 1.**
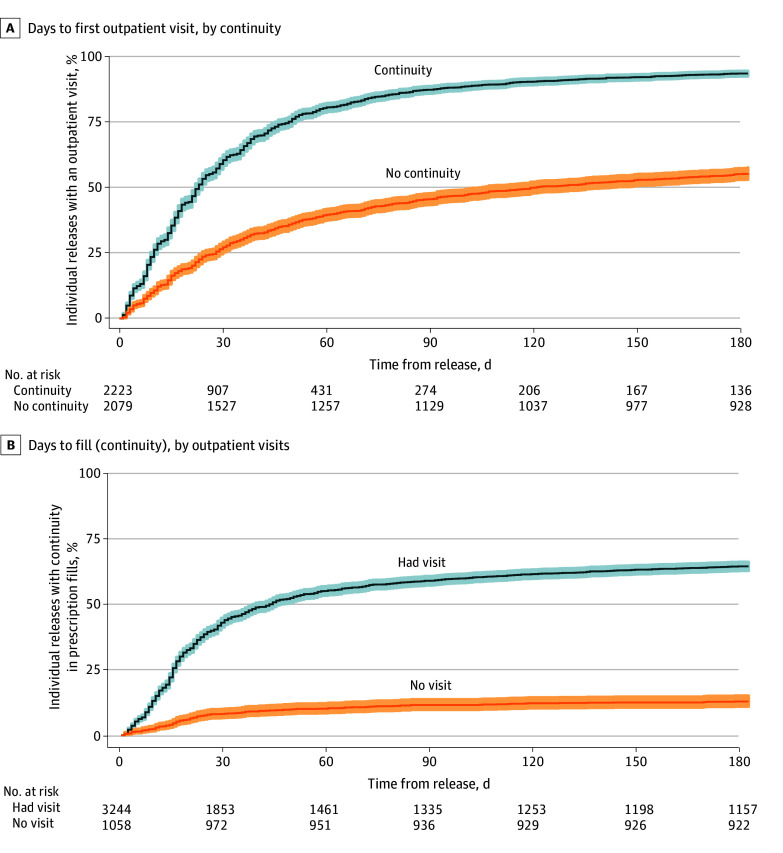
Days to Health Care Use Among the Medicaid-Enrolled Cohort After Release A, Cumulative proportion of the study cohort who had an outpatient visit during the 6 months after release, stratified by prescription continuity. B, Proportion of the cohort who had prescription continuity over the first 6 months after release, stratified by whether they had an outpatient visit in the first 6 months. These figures are the complement of the respective Kaplan-Meier survival curves. Shaded areas represent 95% CIs.

**Figure 2.**
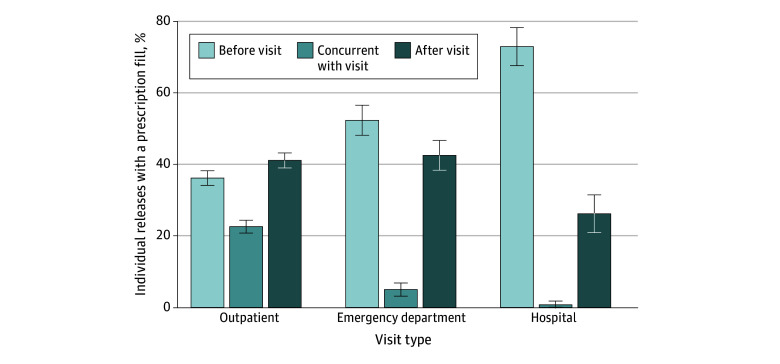
Timing of Prescription Fill Relative to Health Care Visit Distribution of timing of prescription fill relative to health care visit (within 6 months of release) by type of health care use, conditional on having a fill and an outpatient, emergency department, or office visit. Error bars indicate 95% CIs.

Figure 1A illustrates the cumulative proportion of individuals who had an outpatient visit during the 6 months after release, stratified by prescription continuity. Those with continuity had visits more quickly than those who did not. Among those with continuity, 80.6% had a visit within the first 60 days (representing 86.0% of those with continuity who had a visit within the first 6 months), whereas just 39.5% of those without continuity had a visit (72.1% of those who had a visit within the first 6 months). Figure 1B shows the proportion of individuals who had prescription continuity over the first 6 months after release, stratified by whether they had an outpatient visit in the first 6 months. Individuals with no outpatient visit rarely filled prescriptions outside of the first 30 days (indicating likely use of the prescription given to them upon release). Among those who received outpatient care, fill rates were much higher during and after the first 30 days post release. Figure 2 illustrates that among those with a visit, on average, fills occurred concurrently with or following outpatient visits, while we generally observed fills prior to ED and hospital visits. The median time in days between fills and visits was 0 (range, −10 to 9) for outpatient visits, indicative of concurrent receipt, whereas it was 2.5 days (range, −35 to 11) for ED visits and 40 days (range, −98 to 1) for hospitalizations (eTable 5 in Supplement 1).

To facilitate assessment of generalizability, we provide a comparison of the study cohort to the full population of individuals released during the study period in eTable 2 in Supplement 1. We also provide a full description of the most commonly prescribed medications, including OTCs, for both the full population and the study cohort in eTables 3 and 6 in Supplement 1, as well as the fraction of the full population that enrolled in Medicaid by drug class or chronic condition in eTable 7 in Supplement 1.

## Discussion

This study investigated the likelihood of continued prescription fills after incarceration and estimated demographic, carceral, and health care utilization factors associated with medication continuity among Medicaid-insured adults returning to the community from state prison in Wisconsin. Previous studies have shown that adults with a history of incarceration experience high rates of chronic illnesses relative to the general population, including hypertension, diabetes, HIV, and SMI.^3,4,5^ Pharmacotherapy is a core component of effective management of these conditions, and ensuring treatment continuity reduces the risk of disease progression and the occurrence of acute health care events.^6,7,8^ This study examined chronic illness medication continuity among Medicaid-insured adults returning to the community from state prison. Key findings include that (1) prescription continuity rates within 6 months post release ranged from less than 20.0% to 78.7% depending on the condition; (2) outpatient care was associated with continuity and consistently preceded or was concurrent with fills among those with continuity; and (3) continuity varied substantially by age, sex, and race. These findings were unlikely to be driven by insurance status, because individuals were all enrolled in Medicaid at release and equally likely to remain enrolled at 6 months regardless of continuity.

On average, only 51.7% of the study cohort filled a prescription for their chronic illness medication within 6 months of release. Our continuity measure should be interpreted as an upper bound of standard claims measures of medication adherence because we assessed any use within class or condition over an extended time period, meaning these rates were likely lower than adherence rates in the general population.^8^ Understanding the reasons for low continuity and its potential consequences for formerly incarcerated adults’ health and sustained return to the community is a promising area for future research.

In this study, health care use within 6 months of release was the most notable factor associated with continuity. A total of 95.5% of those with prescription continuity had a health care visit, 43.8 percentage points higher than those without continuity. Outpatient visits were associated with higher continuity across medication types and disease classes. At the time of our study, individuals were released with a 2-week supply and a prescription for an additional 30-day supply; the minimum required under Medicaid section 1115 reentry waivers is a 30-day supply. In our study, many individuals did not fill their prescriptions, or receive a new one, unless they also had an outpatient visit early on. These patterns suggest that outpatient visits contribute to continuity, indicating that engaging patients more directly with the health care system immediately following release, such as through case management or primary care interventions, may improve prescription continuity rates.^23^ Further study of the causal role of visits in the timing of prescription fills would aid in understanding the importance of such engagement.

Health policy efforts for justice-involved populations have recently focused on access to insurance.^11^ Medicaid cannot pay for health care services for the incarcerated, but suspension rather than termination of benefits, along with enrollment assistance programs, can improve ongoing insurance enrollment.^10^ This study reveals stark differences in access to care within a relatively homogenous group of recently released individuals enrolled in Medicaid, echoing prior work showing that Black adults leaving prison enroll in Medicaid at rates comparable to White peers^20^ but are less likely to receive outpatient care during the first 30 days,^24^ and are more likely to visit the ED within 6 months,^25^ post release. There are no clear reasons for continuity to vary by condition, race, or sex, suggesting that other factors such as clinician availability, systematic biases, or varying levels of support during reentry may contribute. Understanding these causes is crucial for developing interventions that ensure equitable access to care for all justice-involved individuals.

### Limitations

This study has limitations. The rank of therapeutic classes and continuity in this study may not generalize to other incarcerated populations, due to differences in medication mapping, population health, and health care infrastructure. We may have underestimated continuity because we only observed postrelease health care use paid by Medicaid. Our measure of continuity will be higher than a measure of medication adherence, because it requires only that a prescription be filled and does not consider time to fill or days with medication; it is not a measure of effective chronic disease management. We were unable to assess continuity of medications for opioid use disorder (MOUD) and alcohol use disorder (AUD). The correctional system did not offer MOUD during the study period except through a small naltrexone pilot program; in addition, the number of individuals who received a US Food and Drug Administration–approved medication for treating AUD was fewer than 10, making analysis impractical. Finally, regression results should not be interpreted causally because those who filled prescriptions may differ systematically from those who do not in unobserved ways (eg, may have been less healthy).

## Conclusions

In this cohort study of prescription continuity between prison and the community, continuity was low, even for drugs with high clinical need and relevance for population health. Continuity was especially low among those without outpatient care in the 6 months post incarceration. Understanding ongoing treatment needs of recently incarcerated adults is crucial for crafting interventions that improve health and reduce premature mortality. Our analysis offers estimates of baseline medication continuity during reentry and useful insight on barriers to treatment as health care professionals and policy-makers seek to improve care transitions for this high-risk population. Our findings suggest that effective transitional care interventions may require emphasis on connecting with clinicians alongside Medicaid enrollment assistance and an initial medication supply.

## Notes

Supplement 1. **eMethods 1.** Cohort Construction for Prescription Frequency and Continuity Analysis **eFigure 1.** Flow Chart of Sample Construction Steps **eTable 1.** Characteristics Associated with Medicaid Enrollment Among All Releases Between April 2015 and June 2017 **eMethods 2.** Methodology for Prescription Drug Data Cleaning and Identification **eFigure 2.** Measure Construction Steps **eTable 2.** American Hospital Formulary Service Therapeutic Class to Disease Class Mapping **eTable 3.** Top 25 Drugs Prescribed in the 3 Months Before Release, Medicaid Cohort **eFigure 3.** Difference in Probability of Prescription Continuity by Health Care Usage **eTable 4.** Regression-Adjusted Associations Between Fill and Individual Characteristics, Controlling for or Subsampling to Number of Releases **eTable 5.** Time Between First Postrelease Prescription Medication Fill and First Visit by Type of Visit **eTable 6.** Top 25 Drugs Prescribed in the 3 Months Before Release, All Releases **eTable 7.** Fraction of Releases Enrolling in Medicaid by American Hospital Formulary Service (AHFS) Drug Class

Supplement 2.
Data Sharing Statement
