# Supplementary material for: Continuity of Prescription Medication Use Among Adults Leaving State Prison
Source: JAMA Netw Open. 2025 Feb 27;8(2):e2461982. doi: 10.1001/jamanetworkopen.2024.61982 (PMC11868974; doi:10.1001/jamanetworkopen.2024.61982)
Supplement: Supplement 1. — eMethods 1. Cohort Construction for Prescription Frequency and Continuity Analysis eFigure 1. Flow Chart of Sample Construction Steps eTable 1. Characteristics Associated with Medicaid Enrollment Among All Releases Between April 2015 and June 2017 eMethods 2. Methodology for Prescription Drug Data Cleaning and Identification eFigure 2. Measure Construction Steps eTable 2. American Hospital Formulary Service Therapeutic Class to Disease Class Mapping eTable 3. Top 25 Drugs Prescribed in the 3 Months Before Release, Medicaid Cohort eFigure 3. Difference in Probability of Prescription Continuity by Health Care Usage eTable 4. Regression-Adjusted Associations Between Fill and Individual Characteristics, Controlling for or Subsampling to Number of Releases eTable 5. Time Between First Postrelease Prescription Medication Fill and First Visit by Type of Visit eTable 6. Top 25 Drugs Prescribed in the 3 Months Before Release, All Releases eTable 7. Fraction of Releases Enrolling in Medicaid by American Hospital Formulary Service (AHFS) Drug Class [file jamanetwopen-e2461982-s001.pdf]

Supplementary Online Content

Dague L, McNamara C, Westergaard R, Look KA, Burns M. Continuity of prescription medication use among adults leaving state prison. *JAMA Netw Open*. 2025;8(2):e2461982. doi:10.1001/jamanetworkopen.2024.61982

eMethods 1. Cohort Construction for Prescription Frequency and Continuity Analysis

eFigure 1. Flow Chart of Sample Construction Steps

eTable 1. Characteristics Associated with Medicaid Enrollment Among All Releases Between April 2015 and June 2017

eMethods 2. Methodology for Prescription Drug Data Cleaning and Identification

eFigure 2. Measure Construction Steps

eTable 2. American Hospital Formulary Service Therapeutic Class to Disease Class Mapping

eTable 3. Top 25 Drugs Prescribed in the 3 Months Before Release, Medicaid Cohort

eFigure 3. Difference in Probability of Prescription Continuity by Health Care Usage

eTable 4. Regression-Adjusted Associations Between Fill and Individual Characteristics, Controlling for or Subsampling to Number of Releases

eTable 5. Time Between First Postrelease Prescription Medication Fill and First Visit by Type of Visit

eTable 6. Top 25 Drugs Prescribed in the 3 Months Before Release, All Releases

eTable 7. Fraction of Releases Enrolling in Medicaid by American Hospital Formulary Service (AHFS) Drug Class

This supplementary material has been provided by the authors to give readers additional information about their work.

© 2025 Dague L et al. *JAMA Network Open*.

eMethods 1. Cohort Construction for Prescription Frequency and Continuity Analysis

eFigure 1 shows the steps for the construction of our analysis cohort. We begin with the population of working age adults released from a Wisconsin state correctional facility between April 2015 and June 2017 after an incarceration of at least 31 days for which there are 20,289 releases.

Examining prescription continuity during the transition between prison and the community requires focusing on the subsample of individuals who enroll in Medicaid following their release. We restrict our full sample of 20,844 individuals to those who enroll in Medicaid in the month of release for at least one additional month for which there are 13,792 releases. eTable 1 summarizes the characteristics associated with Medicaid enrollment for our full sample of individuals. Of these, 10,209 receive a prescription in the 3 months preceding release and 4,302 receive a prescription for a prescription drug of interest in the 3 months preceding release.

eFigure 1. Flow Chart of Sample Construction Steps

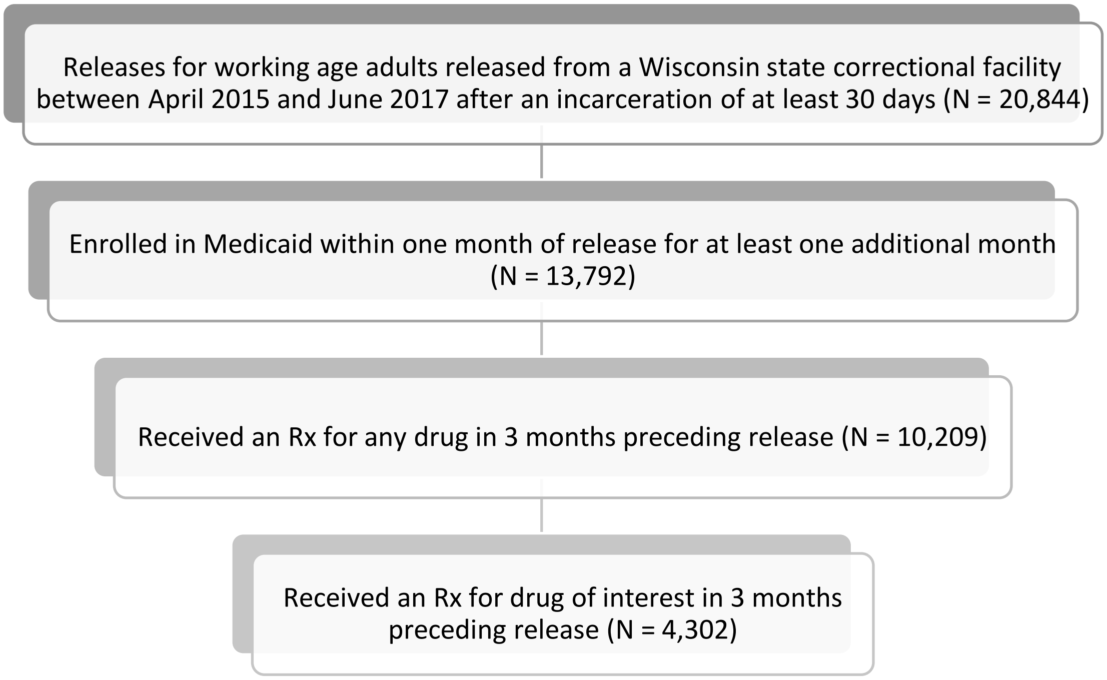

© 2025 Dague L et al. *JAMA Network Open*.

eTable 1. Characteristics Associated with Medicaid Enrollment Among All Releases Between April 2015 and June 2017

	All Releases N=20,844	Does not meet Medicaid cohort criteria N=7,052	Meets Medicaid cohort criteria N=13,792	p-value
Enrolled in Medicaid for 6 months post-release	11,598 (55.6%)	1,715 (24.3%)	9,883 (71.7%)	<0.001
Any outpatient, ER, or hospital visit	8,887 (42.6%)	100 (1.4%)	8,787 (63.7%)	<0.001
Outpatient visit	8,428 (40.4%)	91 (1.3%)	8,337 (60.4%)	<0.001
ER visit	1,955 (9.4%)	47 (0.7%)	1,908 (13.8%)	<0.001
Hospital visit	739 (3.5%)	18 (0.3%)	721 (5.2%)	<0.001
Female	2,008 (9.6%)	554 (7.9%)	1,454 (10.5%)	<0.001
Age released	35.3 (10.7)	34.8 (11.2)	35.5 (10.5)	<0.001
Marital status				<0.001
Single	18,059 (86.6%)	5,876 (83.3%)	12,183 (88.3%)	
Married/partnered	2,072 (9.9%)	860 (12.2%)	1,212 (8.8%)	
Other	713 (3.4%)	316 (4.5%)	397 (2.9%)	
Race				<0.001
Black	8,058 (38.7%)	2,742 (38.9%)	5,316 (38.5%)	
White	11,674 (56.0%)	3,783 (53.6%)	7,891 (57.2%)	
Other	1,112 (5.3%)	527 (7.5%)	585 (4.2%)	
Education				<0.001
< H.S.	5,735 (27.5%)	2,011 (28.5%)	3,724 (27.0%)	
≥ H.S.	14,155 (67.9%)	4,641 (65.8%)	9,514 (69.0%)	
Missing	954 (4.6%)	400 (5.7%)	554 (4.0%)	
Rural status of county of conviction				<0.001
Nonrural	16,730 (80.3%)	5,597 (79.4%)	11,133 (80.7%)	
Rural	3,805 (18.3%)	1,318 (18.7%)	2,487 (18.0%)	
Missing	309 (1.5%)	137 (1.9%)	172 (1.2%)	
Duration of incarceration (months)	25.7 (37.0)	28.1 (43.7)	24.5 (33.0)	<0.001
Security status of release facility				<0.001
Minimum	7,503 (36.0%)	2,611 (37.0%)	4,892 (35.5%)	
Medium	10,736 (51.5%)	3,471 (49.2%)	7,265 (52.7%)	
Medium/maximum	718 (3.4%)	181 (2.6%)	537 (3.9%)	
Maximum	1,716 (8.2%)	680 (9.6%)	1,036 (7.5%)	
Jail	125 (0.6%)	64 (0.9%)	61 (0.4%)	

Sample is all releases for working age adults released from a Wisconsin state correctional facility between April 2015 and June 2017 after an incarceration of at least 31 days (N= 20,844). Medicaid cohort criteria are enrolling in Medicaid within one month of release for at least one additional month. Data are presented as mean (SD) for continuous measures and n (%) for categorical measures. Other race includes American Indian or Alaska Native, Asian or Pacific Islander, and unknown.

© 2025 Dague L et al. *JAMA Network Open*.

eMethods 2. Methodology for Prescription Drug Data Cleaning and Identification

We obtained a list of DOC drug prescriptions for our study cohort. The AHFS therapeutic class description for each drug NDC was identified using Medicaid claims data and added to these data. These data were used to generate a list of all medication names and the number of unique individuals that received each medication during the 3 months pre-release. Our goal was to identify a set of drug classes that have single indications which map to a condition that requires ongoing drug treatment, excluding OTC drugs that may be obtained without a prescription and would not appear in the post-release Medicaid data.

The list was manually reviewed by a licensed pharmacist (Kevin A. Look). Each medication was coded as being available OTC or prescription only. Those drugs that were prescription only were then classified as having a primary indication for the treatment of acute (e.g., pain medications, antibiotics, rescue inhalers, steroids) or chronic conditions. The medications were also reviewed to identify whether they had a single indication or multiple indications (e.g., antidepressants, anticonvulsants, stimulates, etc.). Drugs were considered potential candidates if they were used to treat common chronic conditions requiring ongoing drug treatment and had a single indication and/or limited off-label use.

The list of potential candidate drugs was then reviewed to identify their respective drug classes using the AHFS therapeutic class description. A small number of drugs with missing therapeutic classes were manually screened and coded using their primary AHFS therapeutic class. The most common medications and therapeutic classes were identified by unique individuals. These were used to identify common disease categories of interest (i.e., diabetes, HIV, hypercholesterolemia, and hypertension). The list of potential candidate medications was re-screened to identify the set of all drugs with a primary AHFS therapeutic classes to treat one of the four disease categories of interest. During our study period, medication assisted treatment for opioid use disorder was not offered in the state prison system with the exception of a small naltrexone pilot program and so these drugs do not appear in our candidate lists.

eFigure 2 illustrates the steps taken to construct the potential candidate drugs and identify their drug classes and the effects of those steps on sample size. These steps are also listed below. eTable 2 provides the mapping of AHFS therapeutic classes for potential candidate drugs to disease classes.

1. 841 total medications in original spreadsheet
2. Restricting to “Rx Only” drugs keeps 550 of 841 records (65.4%) – drops 291 observations.
3. Restricting to “Chronic” drugs keeps 290 of 550 records (52.7%) – drops 260 observations.
4. Restricting to “Potential Candidates” keeps 132 of 290 records (45.5%) – drops 158 observations.
5. Final division into 25 therapeutic classes (composed of all 132 drugs) and 4 diseases (composed of 65 drugs)

© 2025 Dague L et al. *JAMA Network Open*.

eFigure 2. Measure Construction Steps

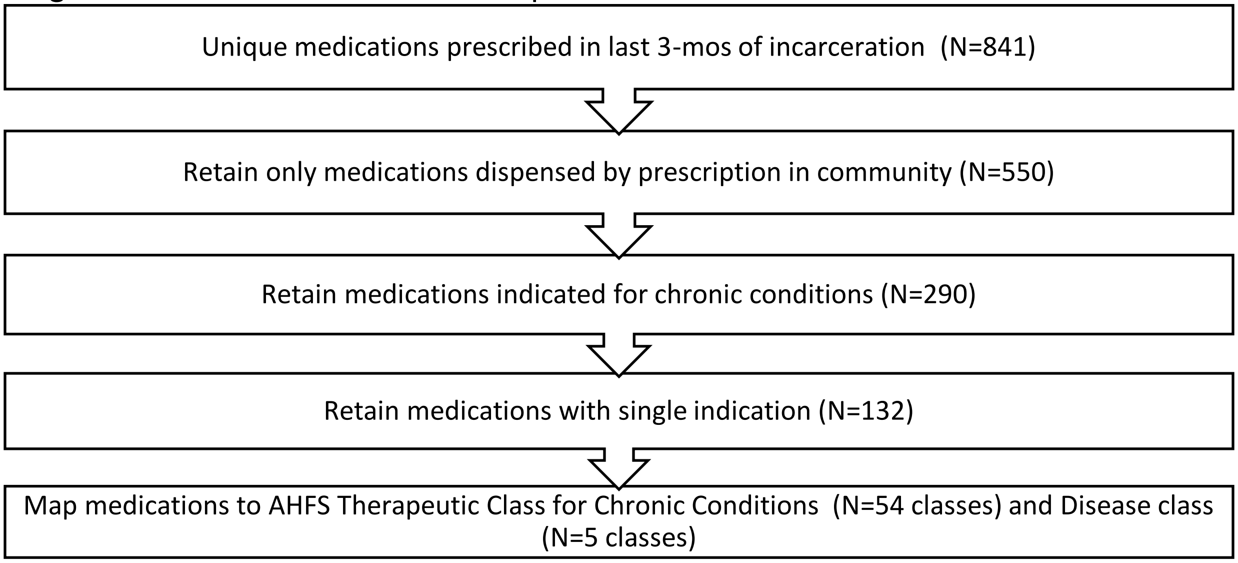

© 2025 Dague L et al. *JAMA Network Open*.

eTable 2. American Hospital Formulary Service Therapeutic Class to Disease Class Mapping

**Disease class**	
Anxiety/Depression	Antidepressants, misc.
	Anxiolytics, sedatives, and hypnotics, misc.
	Benzodiazepines (anticonvulsants)
	Benzodiazepines (anxiolytic, sedative/hyp)
	Selective serotonin norepi reuptake inhibitor
	Selective serotonin reuptake inhibitors
	Serotonin modulators
	Tricyclics, other norepi-ru inhibitors
Diabetes	Alpha-glucosidase inhibitors
	Biguanides
	Dipeptidyl peptidase-4 (dpp4) inhibitors
	Rapid-acting insulins
	Sodium-gluc cotransport 2 (sglt2) inhib
	Sulfonylureas
	Thiazolidinediones
HIV	HIV integrase inhibitor antiretrovirals
	HIV nonnucleoside rev.transcrip. inhib.
	HIV nucleoside, nucleotide rt inhibitors
	HIV protease inhibitor antiretrovirals
Hypercholesterolemia	Cholesterol absorption inhibitors
	Fibric acid derivatives
	HMG-COA reductase inhibitors
Hypertension	Alpha-adrenergic blocking agents
	Angiotensin ii receptor antagonists
	Angiotensin-converting enzyme inhibitors
	Beta-adrenergic blocking agents
	Calcium-channel blocking agents, misc.
	Dihydropyridines
	Direct vasodilators
	Potassium-sparing diuretics
	Thiazide diuretics
	Thiazide-like diuretics

© 2025 Dague L et al. *JAMA Network Open*.

eTable 3. Top 25 Drugs Prescribed in the 3 Months Before Release, Medicaid Cohort

Rank	(1) All drugs		(2) Single-indication prescription drugs for chronic conditions	
	Name	Number (%) of individuals with Rx	Name	Number (%) of individuals with Rx
1	Ibuprofen	5361 (0.55)	Lisinopril	1041 (0.11)
2	Acetaminophen	3171 (0.31)	Hydrochlorothiazide	968 (0.1)
3	Naproxen	2180 (0.22)	Simvastatin	755 (0.08)
4	Amoxicillin	1703 (0.18)	Risperidone	637 (0.06)
5	Diphenhydramine	1532 (0.15)	Clonidine	540 (0.05)
6	Penicillin	1443 (0.14)	Metoprolol	472 (0.05)
7	Albuterol	1439 (0.15)	Metformin	427 (0.04)
8	Trazodone	1423 (0.13)	Amlodipine	424 (0.04)
9	Gabapentin	1288 (0.12)	Ciclesonide	368 (0.04)
10	Mirtazapine	1186 (0.12)	Lithium	354 (0.03)
11	Omeprazole	1122 (0.11)	Levothyroxine	342 (0.03)
12	Lisinopril	1041 (0.11)	Quetiapine	309 (0.03)
13	Sertraline	1024 (0.1)	Insulin	299 (0.03)
14	Eucerin/Minerin	1004 (0.1)	Atorvastatin	296 (0.03)
15	Hydrochlorothiazide	968 (0.1)	Propranolol	248 (0.02)
16	Triamcin	948 (0.1)	Ziprasidone	234 (0.02)
17	Buspirone	930 (0.09)	Benztropine	204 (0.02)
18	Fluoxetine	916 (0.09)	Haloperidol	188 (0.02)
19	Hydroxyzine	903 (0.09)	Losartan	182 (0.02)
20	Ranitidine	903 (0.09)	Mometasone	173 (0.02)
21	Hydrocodone/Apap	888 (0.09)	Olanzapine	152 (0.01)
22	Aspirin	875 (0.09)	Montelukast	142 (0.01)
23	Venlafaxine	826 (0.08)	Pravastatin	141 (0.01)
24	Clindamycin	823 (0.08)	Atenolol	137 (0.01)
25	Loratadine	812 (0.08)	Oxybutynin	129 (0.01)
	Any Rx	10209 (0.74)	Any Rx meeting criteria	4302 (0.32)

Notes: Includes prescriptions written within 3 months of release for all releases from April 2015 to June 2017 who enroll in Medicaid in the month of release for at least one additional month. Share denominator is number of individual releases from a Wisconsin state correctional facility between April 2015-June 2017 who enroll in Medicaid in the month of release for at least one additional month, of which there were 13,792.

© 2025 Dague L et al. *JAMA Network Open*.

eFigure 3. Difference in Probability of Prescription Continuity by Health Care Usage

Panel A. By Any Outpatient Visit in First Six Months Post-Release

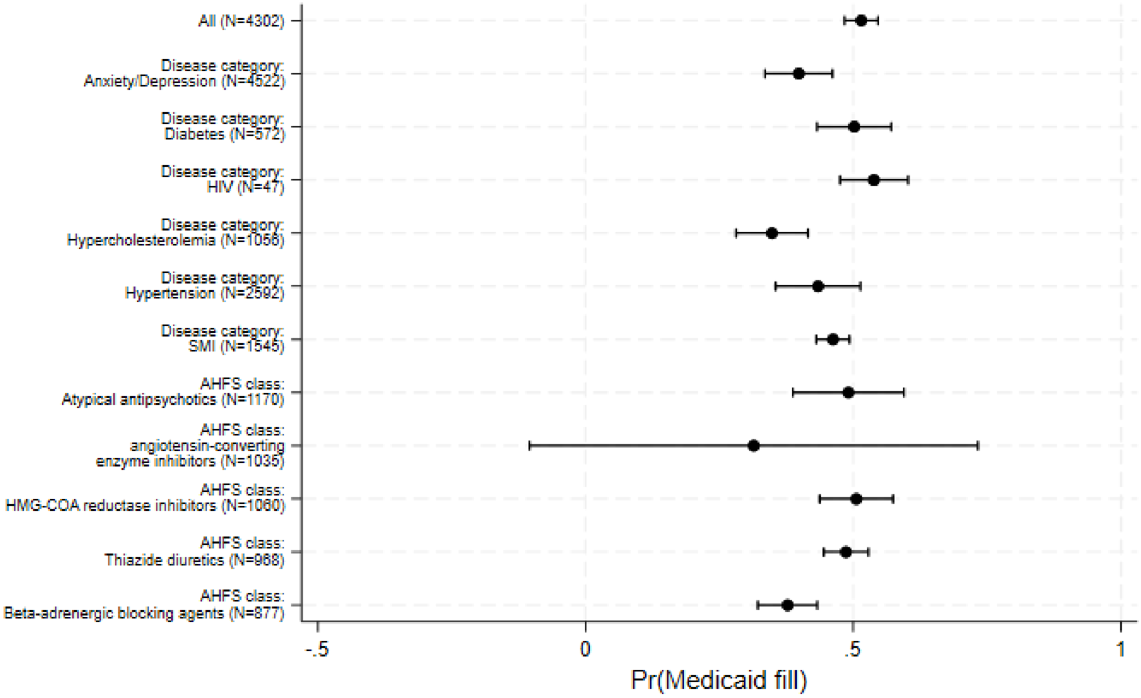

Panel B. By Any Emergency Visit in First Six Months Post-Release

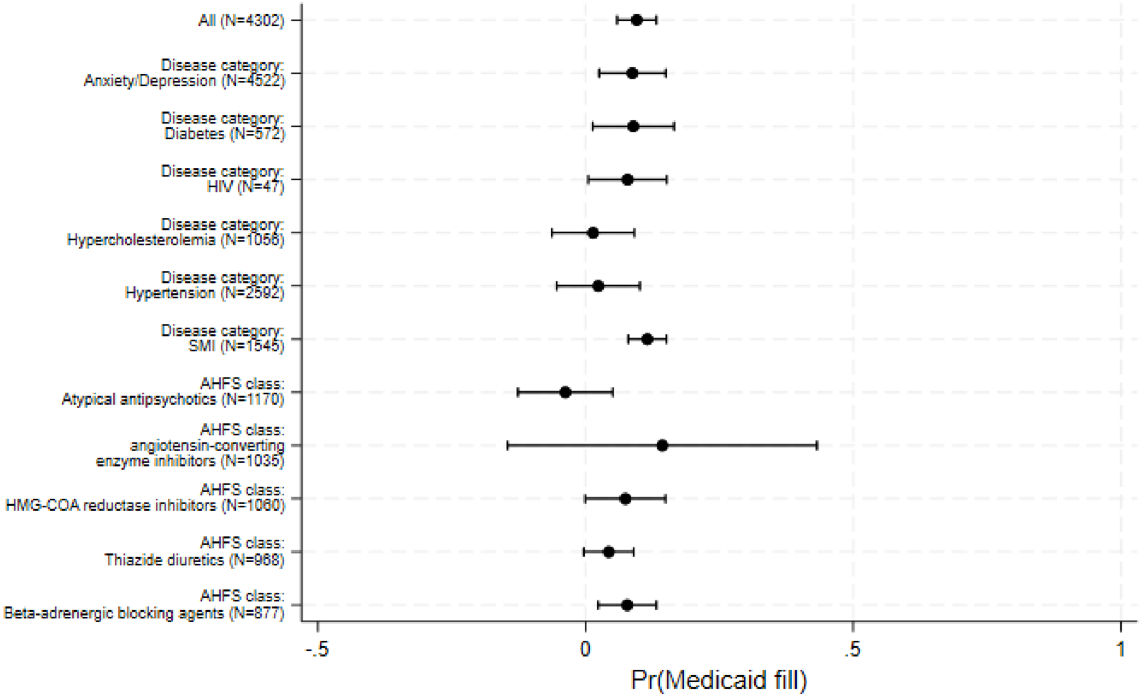

© 2025 Dague L et al. *JAMA Network Open*.

Panel C. By Any Hospitalization in First Six Months Post-Release

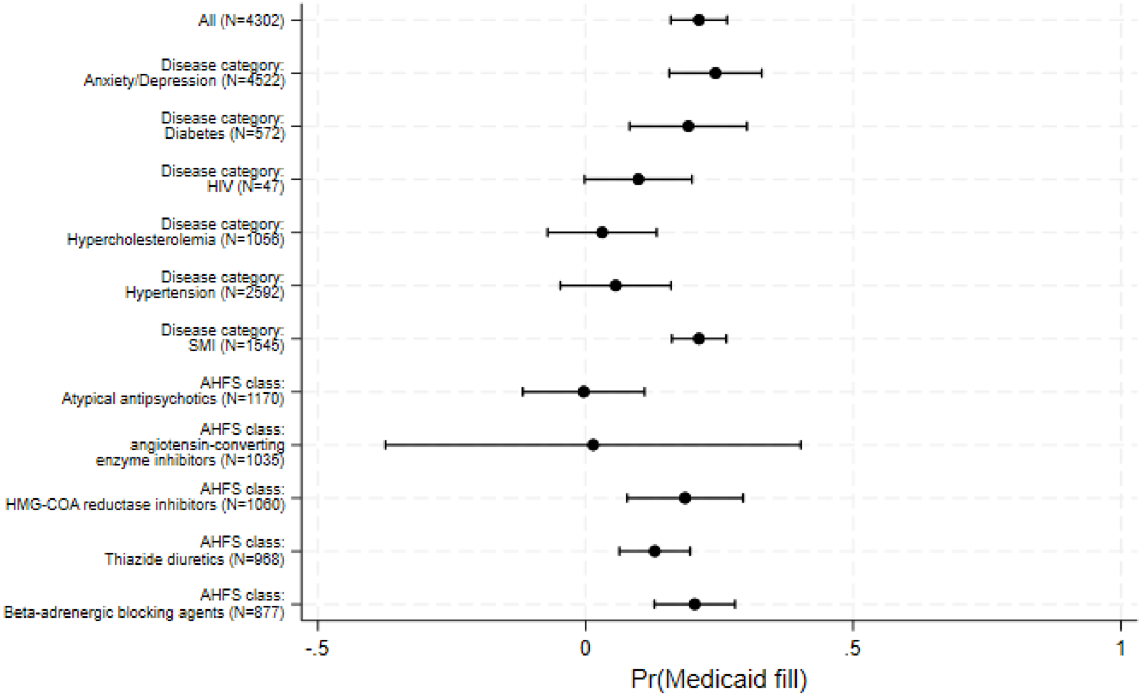

Notes: Includes prescriptions written within 3 months of release for all releases from April 2015 to June 2017 who enroll in Medicaid in the month of release for at least one additional month. Share denominator is number of individual releases from a Wisconsin state correctional facility between April 2015-June 2017 who enroll in Medicaid in the month of release for at least one additional month, of which there were 13,792.

© 2025 Dague L et al. *JAMA Network Open*.

eTable 4. Regression-Adjusted Associations Between Fill and Individual Characteristics, Controlling for or Subsampling to Number of Releases

	Percentage point difference relative to baseline (95% CI)			
	**(1) Individual releases with a fill in any class during the 3 months before release**		**(2) Individuals with a fill in any class during the 3 months before release, and at most one release during sample period**	
Greater than 1 release	2.5	[-1.9,7.0]		
Enrolled in Medicaid for ^{3} 6 months post-release	0.8	[-2.2,3.7]	0.6	[-2.5,3.7]
Health care use within 6 months post release				
Outpatient visit	43.9	[40.9,47.0]	44.9	[41.5,48.3]
ER visit	2.3	[-1.1,5.7]	1.3	[-2.4,4.9]
Hospital visit	9.9	[5.0,14.8]	9.6	[4.3,15.0]
Female	11.0	[5.7,16.2]	10.8	[5.4,16.2]
Age released	0.9	[0.7,1.0]	0.9	[0.7,1.0]
Marital status				
Single	(ref)		(ref)	
Married/partnered	4.0	[-0.2,8.2]	4.1	[-0.3,8.5]
Other	-13.8	[-28.7,1.0]	-7.4	[-26.6,11.8]
Race				
White	(ref)		(ref)	
Black	-0.5	[-3.5,2.5]	-0.5	[-3.7,2.7]
Other	-11.6	[-18.4,-4.7]	-10.8	[-18.0,-3.7]
Education				
< H.S.	(ref)		(ref)	
≥ H.S.	-0.5	[-3.6,2.6]	-0.4	[-3.7,2.9]
Missing	2.9	[-8.3,14.0]	1.7	[-11.1,14.5]
Rural status of county of conviction				
Nonrural	(ref)		(ref)	
Rural	0.2	[-3.2,3.6]	0.8	[-2.8,4.4]
Missing	6.8	[-4.1,17.6]	3.0	[-9.7,15.7]
Duration of incarceration (months)	0.0	[-0.0,0.1]	0.0	[0.0,0.1]
Security status of release facility				
Minimum	-1.3	[-4.3,1.8]	(ref)	
Medium	-3.8	[-10.8,3.1]	-1.0	[-4.2,2.2]
Medium/maximum	-2.9	[-8.1,2.3]	-4.1	[-11.4,3.2]
Maximum	-6.9	[-47.0,33.1]	-1.3	[-7.0,4.4]
Jail	-1.3	[-4.3,1.8]	-5.8	[-45.1,33.6]
Observations	4,302		3,804	

Notes: Results of logistic regression of indicator for prescription continuity on individual characteristics. Cells contain marginal effects*100 and 95% confidence intervals in brackets. Standard errors are clustered at the individual level. Sample for results reported in Column (1) is all individual releases between April 2015 and June 2017 who had at least one prescription for a drug in one of the drug classes of interest from table 1 in the three months preceding release and who were enrolled in Medicare in the month of release (N= 4,302). Sample for results reported in Column (2) is the same as Column (1) but restricted to individuals with at most one release during the sample period.

eTable 5. Time Between First Postrelease Prescription Medication Fill and First Visit by Type of Visit

Visit type	N	Mean	10th pctile	25th pctile	Median	75th pctile	90th pctile
Outpatient	2087	0.82	-33	-10	0	9	32
ED	540	-13.48	-101	-35	-2.5	11	44.5
Hospital	267	-46.26	-133	-98	-40	1	24

Notes. Table includes Medicaid cohort members who received a Medicaid prescription medication fill for at least one single-indication drug for a chronic condition within six months of their release and had a visit within six months of their release. The table shows the distribution of time in days between first Medicaid fill and first visit. Positive values indicate health care use that occurred prior to the fill and negative values indicate health care use following the fill.

© 2025 Dague L et al. *JAMA Network Open*.

To facilitate assessment of generalizability, we include in eTable 6, the pre-release prescription medications for the 20,844 releases observed during the study period; this table is analogous to eTable 3 for the study cohort. Among the 20,844 releases, 14,653 received an Rx for some drug in the 3 months preceding release. Column (1) summarizes prescription frequency in the 3 months preceding release for these 14,653 releases. Column (2) summarizes prescription frequency in the 3 months preceding release for individuals who receive an Rx for a prescription drug of interest in the 3 months preceding release, for which there are 6,012 releases.

eTable 6. Top 25 Drugs Prescribed in the 3 Months Before Release, All Releases

Rank	(1) All drugs		(2) Single-indication prescription drugs for chronic conditions	
	Name	Number (%) of individuals with Rx	Name	Number (%) of individuals with Rx
1	Ibuprofen	7555 (0.36)	Lisinopril	1516 (0.07)
2	Acetaminophen	4311 (0.21)	Hydrochlorothiazide	1355 (0.06)
3	Naproxen	3051 (0.15)	Simvastatin	1101 (0.05)
4	Amoxicillin	2422 (0.12)	Risperidone	849 (0.04)
5	Diphenhydramine	2044 (0.1)	Clonidine	689 (0.03)
6	Albuterol	2022 (0.1)	Metoprolol	672 (0.03)
7	Penicillin	1971 (0.09)	Metformin	614 (0.03)
8	Trazodone	1858 (0.09)	Amlodipine	597 (0.03)
9	Gabapentin	1690 (0.08)	Ciclesonide	500 (0.02)
10	Mirtazapine	1589 (0.08)	Levothyroxine	473 (0.02)
11	Omeprazole	1537 (0.07)	Lithium	438 (0.02)
12	Lisinopril	1516 (0.07)	Quetiapine	432 (0.02)
13	Eucerin/Minerin	1369 (0.07)	Atorvastatin	413 (0.02)
14	Sertraline	1361 (0.07)	Insulin	410 (0.02)
15	Triamcin	1355 (0.06)	Propranolol	340 (0.02)
16	Hydrochlorothiazide	1355 (0.06)	Ziprasidone	321 (0.02)
17	Aspirin	1234 (0.06)	Benztropine	281 (0.01)
18	Ranitidine	1230 (0.06)	Haloperidol	269 (0.01)
19	Fluoxetine	1197 (0.06)	Mometasone	254 (0.01)
20	Hydrocodone/Apap	1197 (0.06)	Losartan	243 (0.01)
21	Hydroxyzine	1191 (0.06)	Olanzapine	198 (0.01)
22	Buspirone	1190 (0.06)	Pravastatin	197 (0.01)
23	Loratadine	1147 (0.06)	Chlorthalidone	189 (0.01)
24	Clindamycin	1131 (0.05)	Montelukast	189 (0.01)
25	Meloxicam	1118 (0.05)	Atenolol	188 (0.01)
	Any Rx	14653 (0.70)	Any Rx meeting criteria	6012 (0.29)

Notes: Includes prescriptions written within 3 months of release for all releases from April 2015 to June 2017. Share denominator is number of individual releases from a Wisconsin state correctional facility between April 2015-June 2017, of which there were 20,844.

eTable 7. Fraction of Releases Enrolling in Medicaid by American Hospital Formulary Service (AHFS) Drug Class

		(1)	(2)
**Rank**	**AHFS drug class**	**Number (%) of individual-releases with fill in class in 3 months preceding release**	**Of (1), number (%) who enroll in Medicaid in month of release**
1	Atypical antipsychotics	1578 (0.08)	1170 (0.74)
2	Angiotensin-converting enzyme inhibitors	1550 (0.07)	1060 (0.68)
3	HMG-COA reductase inhibitors	1491 (0.07)	1035 (0.69)
4	Thiazide diuretics	1355 (0.06)	968 (0.71)
5	Beta-adrenergic blocking agents	1229 (0.06)	877 (0.71)
6	Corticosteroids (EENT)	836 (0.04)	602 (0.72)
7	Central alpha-agonists	689 (0.03)	540 (0.78)
8	Biguanides	614 (0.03)	427 (0.7)
9	Dihydropyridines	597 (0.03)	424 (0.71)
10	Thyroid agents	473 (0.02)	342 (0.72)
11	Antimanic agents	438 (0.02)	354 (0.81)
12	Rapid-acting insulins	410 (0.02)	299 (0.73)
13	Butyrophenones	283 (0.01)	200 (0.71)
14	Anticholinergic agents (CNS)	281 (0.01)	204 (0.73)
15	Sulfonylureas	249 (0.01)	176 (0.71)
16	Angiotensin II receptor antagonists	248 (0.01)	185 (0.75)
17	Antimuscarinics	192 (0.01)	135 (0.70)
18	Leukotriene modifiers	189 (0.01)	142 (0.75)
19	Thiazide-like diuretics	189 (0.01)	128 (0.68)
20	Calcium-channel blocking agents, misc.	168 (0.01)	120 (0.71)
21	Antimuscarinics/antispasmodics	116 (0.01)	83 (0.72)
22	Fibric acid derivatives	91 (<0.01)	71 (0.78)
23	Antigout agents	90 (<0.01)	65 (0.72)
24	Alpha-adrenergic blocking agents	73 (<0.01)	53 (0.73)
25	EENT drugs, miscellaneous	72 (<0.01)	49 (0.68)
	**Any Rx in AHFS drug class of interest**	**6012 (0.29)**	**4302 (0.72)**
**Disease class**		**(1)**	**(2)**
		**Number (%) of individual-releases with fill in class in 3 mos preceding release**	**Of (1), number (%) who enroll in Medicaid in month of release**
Anxiety/Depression		6308 (0.30)	4522 (0.72)
Hypertension		3606 (0.17)	2592 (0.72)
Severe Mental Illness (SMI)		2050 (0.10)	1545 (0.75)
Hypercholesterolemia		1525 (0.07)	1056 (0.69)
Diabetes		806 (0.04)	572 (0.71)
Human Immunodeficiency Virus (HIV)		69 (<0.01)	47 (0.68)

© 2025 Dague L et al. *JAMA Network Open*.

Notes: For each drug class, column (1) of table 2 shows the number of individual releases from a Wisconsin state correctional facility between April 2015 and June 2017 (N=20,844) who received a prescription for a drug in the corresponding class in the 3 months preceding release. Column (2) shows the share of individuals from column (1) who enrolled in Medicaid in the month of release for at least one month, of which there are 13,792 across all drug classes.

© 2025 Dague L et al. *JAMA Network Open*.
