# Supplementary material for: Continuity of Prescription Medication Use Among Adults Leaving State Prison
Source: JAMA Netw Open. 2025 Feb 27;8(2):e2461982. doi: 10.1001/jamanetworkopen.2024.61982 (PMC11868974; doi:10.1001/jamanetworkopen.2024.61982)
Supplement: Supplement 2. — Data Sharing Statement [file jamanetwopen-e2461982-s002.pdf]

Data Sharing Statement

Dague. Continuity of Prescription Medication Use Among Adults Leaving State Prison. *JAMA Netw Open*. Published February 27, 2025. doi:10.1001/jamanetworkopen.2024.61982

Data
Data available: No

Additional Information
Explanation for why data not available: The data use agreements between the agencies providing data to the university does not permit sharing of data.
